# Effect of Maternal Sildenafil Supplementation During Gestation on the Reproductive Performance of Sows/Gilts and Growth Performance of Neonatal Piglets

**DOI:** 10.3389/fvets.2022.875810

**Published:** 2022-07-22

**Authors:** Yue Wang, Yusheng Qin, Wei Zhao, Fusheng Yao, Wenjing Wang, Xiao Hu, Linghua Cheng, Lei An, Jianhui Tian

**Affiliations:** ^1^Key Laboratory of Animal Genetics, Breeding and Reproduction of the Ministry of Agriculture and Rural Affairs, National Engineering Laboratory for Animal Breeding, College of Animal Science and Technology, China Agricultural University, Beijing, China; ^2^Institute of Animal Husbandry and Veterinary Medicine, Beijing Academy of Agriculture and Forestry Sciences, Beijing, China

**Keywords:** sildenafil, sows, gilts, litter size, birth weight

## Abstract

Sildenafil, a phosphodiesterase 5 (PDE-5) inhibitor, provides an alternative and effective strategy to increase uterine blood flow and vascular development, thus improving fetal development. Previous studies have shown that sildenafil attenuates fetal growth restriction in rodents, sheep, and humans. However, the effect of sildenafil intervention on fetal growth in pigs remains unclear. This study aims to evaluate the effect of dietary supplementation with sildenafil on the reproductive performance of sows and gilts. Over 700 Landrace × Large White crossbred sows in the 3rd or 4th parity were randomly assigned to the control group and the sildenafil treatment groups. In brief, sows in the treatment groups were given a basic diet supplemented with sildenafil (0.32 g/day) during different stages of gestation: (1) gestation day (GD) 0–110; (2) GD 0–30; (3) GD 30–80; (4) GD 80–110; and (5) GD 30–110. At parturition, the total number of piglets born per litter, the number of piglets born alive or dead, the average birth weight, the number of weaned piglets, and the average weaning weights were recorded and analyzed. Sildenafil supplementation throughout gestation (GD 0–110) increased both the litter size and the average birth weight. To reduce the cost of extended sildenafil supplementation, time-phased strategies were further tested. Sildenafil supplementation during early gestation (GD 0–30), mid-gestation (GD 30–80), and late gestation (GD 80–110) partially improved litter performance. Notably, sows fed sildenafil supplementation from the mid-to-late gestation period (GD 30–110) showed significantly improved litter performance, approaching the levels in the sows supplemented with sildenafil throughout the whole gestation period. Taken together, our results showed that maternal sildenafil supplementation during gestation can effectively improve the reproductive performance of sows and gilts, and enhance the growth performance of neonatal piglets, thus providing a promising and practical intervention strategy to improve reproductive management in pig farming.

## Introduction

The production of piglets by sows significantly influences the economic benefits of pig farming. However, the reproductive performance of sows is frequently unsatisfactory because of many environmental, nutritional, and pathological factors (1–3). In particular, the maternal uterine status during gestation is essential for good reproductive performance, such as the litter size, the number of piglets born alive or dead, as well as the growth performance of neonatal piglets, including their birth weight and weaning weight (4–7). In addition, suboptimal uterine status has been reported to be highly associated with the occurrence of birth defects and stillbirths. Thus, intervention to improve the maternal uterine status is a potential strategy to enhance reproductive performance and neonatal growth.

Sildenafil, a phosphodiesterase 5 (PDE-5) inhibitor that blocks cyclic guanosine monophosphate (cGMP) hydrolysis, thereby enhancing nitric oxide (NO)-dependent vasodilatation. It provides an alternative and effective strategy for improving the uterine status and fetal growth, because increased uterine cGMP levels can increase uterine blood flow and vascular development (8). In addition to evidence demonstrating the efficacy of sildenafil in improving fetal growth, particularly in rodents (9–12), clinical reports also showed that sildenafil could improve uterine blood flow, alleviate severe early-onset intrauterine growth restriction, and improve maternal blood pressure regulation during preeclampsia pregnancies (13–17). Long-term administration of sildenafil citrate enhanced fetal weight in both adequately fed and nutrient-restricted ewes (18). However, the efficacy of sildenafil intervention in terms of fetal growth in sows or gilts remains undetermined.

In the present study, we evaluated the efficacy of dietary sildenafil supplementation during gestation in terms of improving the reproductive performance of sows and gilts. We designed a series of experiments in which diets were supplemented with sildenafil at various stages of gestation. The results indicated that maternal sildenafil supplementation during gestation can effectively improve the reproductive outcomes of sows and gilts, and enhance the growth performance of neonatal piglets, thus providing a promising and practical intervention strategy to improve reproductive management in pig farming.

## Materials and Methods

### Animals and Treatments

**Figure 1:**
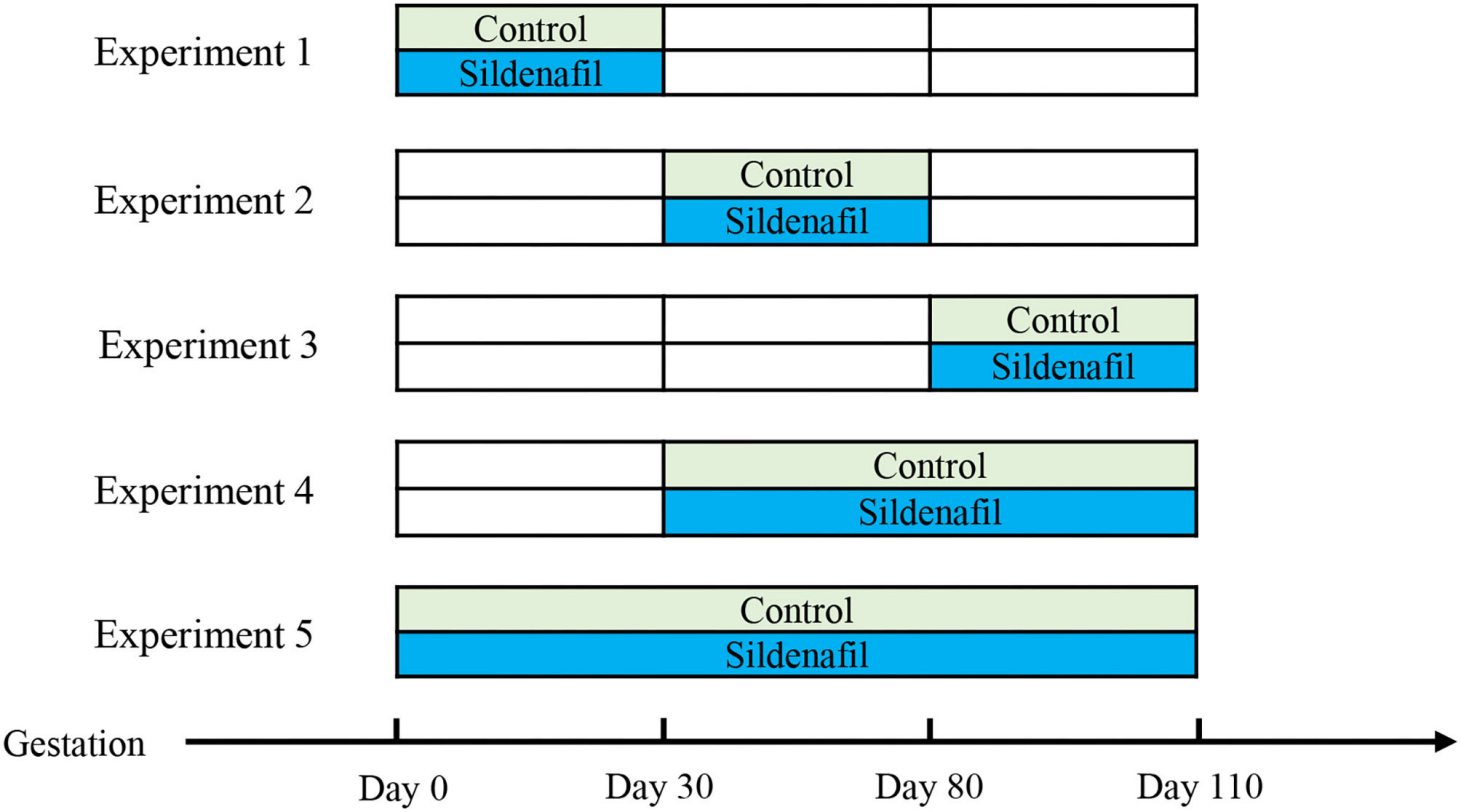
Schematic representation of the experimental design based on the different timing of sildenafil supplementation during gestation.

This study was performed between June and November. More than 700 Landrace × Large White cross-bred sows with an initial body weight of 138.23 ± 6.34 kg were used in this study. All animals (sows, gilts, and piglets) were raised by Muyuan Food Co., Ltd. (Henan, China), which uses a large-scale intensive raising model. The animals were housed under uniform feeding and housing conditions. The 2–3-year-old sows in the 3rd and 4th parity, as well as 230-day-old gilts with weights ranging from 120 to 130 kg, were randomly divided into control or treatment groups (Figure 1). The sows in the control group fed with a standard grain-based diet. The sows in the sildenafil-supplemented group were given a basic diet supplemented with sildenafil (0.32 g/day; Wuhan Remote Co-creation Technology Co., LTD, Wuhan, China) (19) during different stages of gestation: (1) GD 0–110; (2) GD 0–30; (3) GD 30–80; (4) GD 80–110; and (5) GD 30–110. During the afternoon feed, 5 ml of soy oil containing 0.32 g of sildenafil or not was administered to sows using a veterinary filling gun applicator.

### Data Collection

Sows were transferred into the farrowing units, 1 week before the expected date of parturition. The sows were raised in a warm and quiet environment, and any abnormal behavior was recorded. At farrowing, litters with no piglets born alive were excluded. Immediately after completion of farrowing, the numbers of total piglets born and born alive were registered for each sow. In addition, the numbers of expelled mummified piglets (mummies) and stillborn piglets were also determined. Within 24 h of farrowing, after initial colostrum consumption, each individual live piglet was identified with an inimitable ear tag and weighed using electronic scales. The individual body weights of the piglets were recorded. Data from these piglets were used to derive the litter traits of the sow, such as the total number of born piglets, total litter birth weight, and piglet average birth weight.

This study involved 4,076 and 5,193 piglets born alive from the control and sildenafil groups, respectively. About 10 days after birth, all piglets received creep feed during the last week of the lactation period to acclimatize them to dry food. At weaning, performed at around 27 days old, each piglet that survived the suckling period was weighted using electronic scales to record piglet weaning weights. The litter weaning weight and piglet average weaning weight were calculated according to the individual piglet weaning weights.

### Statistical Analysis

All data analyses were conducted using the Statistical Program for Social Sciences, SPSS 20.0 (IBM Corp., Armonk, NY, USA). Individual sows served as the experimental unit. The least significant difference (LSD) test was used to compare the least-squares means. The pooled standard deviation (SD) was calculated for each measurement. A probability of *p* < 0.05 was considered significant, and 0.05 < *p* < 0.1 was declared as a trend. For litter performance, the data of the litter weight, the average piglet weight at birth and weaning, and the average daily gain of piglets were analyzed using the default normal linear regression model. Count data, such as the number of total piglets born, born alive, after cross-fostering, death, and weaned piglets, were analyzed using a Poisson regression model, while a zero-inflated Poisson regression model was used to analyze the numbers of stillborns and mummies. Furthermore, a binomial regression model was used to analyze preweaning piglet mortality.

## Results

### Effect of Sildenafil Intervention From Gestation day 0 to 110 on the Litter Performance of Sows

To determine whether dietary sildenafil supplementation throughout the whole gestation period of sows has a beneficial effect on litter performance, 148 sows were fed the sildenafil diet from GD 0 to GD 110. At farrowing, sows in the sildenafil-supplemented group gave birth to more live piglets than those in the control group. In addition, the sildenafil-supplemented group had a greater litter birth weight and showed a significant increase in the average birth weight of the piglets born alive (Table 1).

**Table 1:** Effect of sildenafil intervention from day 0 to 110 of gestation on sow litter performance (means ± SD).

**Item**	**Control**	**Sildenafil**
No. of litters	62	148
Piglets born, no./litter	10.90 ± 2.27	11.65 ± 2.01^*^
Piglets born alive, no./litter	10.77 ± 2.16	11.52 ± 1.99^*^
Stillborn, no./litter	0.15	0.1
Mummies, no./litter	0.02	0.03
Litter birth weight, kg	15.03 ± 3.14	16.54 ± 2.72^*^
Piglet average birth weight, kg	1.36 ± 0.20	1.46 ± 0.22^*^

* *p < 0.05*.

### Effect of Sildenafil Intervention From Gestation day 0 to 30 on the Litter Performance of Sows

To reduce the cost of prolonged sildenafil supplementation, we designed a series of time-phased strategies and tested the litter performance of sows. First, we assessed the effect of sildenafil intervention on reproductive performance and neonatal growth during the first trimester of gestation. Then, 46 sows were given the basic diet supplemented with sildenafil (0.32 g/day) from GD 0 to GD 30. At farrowing, the total number of born piglets and the litter birth weight were higher in the sildenafil supplementation group than in the control group. It should be mentioned that GD 0–30 sildenafil intervention tended to increase the number of piglets born alive, although the difference was not statistically significant (Table 2). Collectively, these results indicate that dietary sildenafil supplementation during early gestation of sows has beneficial effects on the total number of piglets born and the litter birth weight.

**Table 2:** Effect of sildenafil intervention from day 0 to 30 of gestation on sow litter performance (means ± SD).

**Item**	**Control**	**Sildenafil**
No. of litters	43	46
Piglets born, no./litter	10.63 ± 2.04	11.43 ± 1.64^*^
Piglets born alive, no./litter	10.44 ± 2.00	11.17 ± 1.55
Stillborn, no./litter	0.21	0.26
Mummies, no./litter	0.05	0
Litter birth weight, kg	15.35 ± 3.55	16.66 ± 2.31^*^
Piglet average birth weight, kg	1.48 ± 0.41	1.48 ± 0.29

* *p < 0.05*.

### Effect of Sildenafil Intervention From Gestation day 30 to 80 on the Litter Performance of Sows

To further explore the effect of sildenafil intervention during mid-gestation on litter performance, 76 sows were given the basic diet supplemented with sildenafil (0.32 g/day) from GD 30 to GD 80. At farrowing, the total number of piglets born, the number of piglets born alive, and the number of weaned piglets were significantly higher in the sildenafil group than in the control group. In addition, sildenafil supplementation significantly increased the litter birth weight. Compared with those in the control group, stillbirths, mummies, and weak births were not affected by sildenafil supplementation (Table 3). These results showed that dietary sildenafil supplementation during mid-gestation of sows has beneficial effects on the litter birth weight and piglets born alive, but not on the total number of piglets born.

**Table 3:** Effect of sildenafil intervention from day 30 to 80 of gestation on sow litter performance (means ± SD).

**Item**	**Control**	**Sildenafil**
No. of litters	135	76
Piglets born, no./litter	10.78 ± 2.34	11.63 ± 2.08^*^
Piglets born alive, no./litter	10.48 ± 2.27	11.51 ± 2.02^*^
Piglets weaned, no./litter	9.36 ± 1.72	10.58 ± 1.64^*^
Stillborn, no./litter	0.28	0.1
Mummies, no./litter	0.04	0.01
Litter birth weight, kg	15.56 ± 3.79	17.07 ± 3.05^*^
Piglet average birth weight, kg	1.46 ± 0.27	1.49 ± 0.23
Litter weaning weight, kg	53.71 ± 13.68	57.16 ± 13.07
Piglet average weaning weight, kg	5.73 ± 1.09	5.48 ± 1.18

* *p < 0.05*.

### Effect of Sildenafil Intervention From Gestation day 80 to 110 on the Litter Performance of Sows

To further investigate the effect of sildenafil intervention in the last trimester of gestation on litter performance, 116 sows were given the basic diet supplemented with sildenafil (0.32 g/day) from GD 80 to GD 110. Sildenafil intervention during late gestation significantly increased the litter birth weight and the piglet birth weight, but not the number of piglets born (Table 4).

**Table 4:** Effect of sildenafil intervention from day 80 to 110 of gestation on sow litter performance (means ± SD).

**Item**	**Control**	**Sildenafil**
No. of litters	75	116
Piglets born, no./litter	11.44 ± 2.53	11.36 ± 2.38
Piglets born alive, no./litter	11.29 ± 2.51	11.10 ± 2.29
Stillborn, no./litter	0.13	0.27
Mummies, no./litter	0.01	0
Litter birth weight, kg	17.24 ± 3.61	18.34 ± 3.41^*^
Piglet average birth weight, kg	1.54 ± 0.23	1.67 ± 0.24^*^

* *p < 0.05*.

### Effect of Sildenafil Intervention From Gestation day 30 to 110 on the Litter Performance of Sows

Having confirmed the effect of dietary supplementation of sildenafil on sow litter performance at the early-, mid-, and late-gestation periods, we next attempted to further confirm the effects of dietary sildenafil supplementation on sow litter performance during mid-to-late gestation period. In total, 51 sows were given the basic diet supplemented with sildenafil (0.32 g/day) from GD 30 to GD 110. There was no difference in the piglet birth weight between the sildenafil group and the control group. However, sildenafil supplementation significantly increased the number of piglets born alive, as well as the number of piglets weaned. This advantage in piglets born alive continued through the weaning age at 28 days (*p* < 0.05). Our results indicated that dietary sildenafil supplementation during mid-to-late gestation of sows significantly increased the litter birth weight, without reducing the piglet average birth weight (Table 5). Taken together, the above results suggest that dietary sildenafil supplementation during mid-to-late gestation is an economical and effective strategy to improve the reproductive performance of sows.

**Table 5:** Effect of sildenafil intervention from day 30 to 110 of gestation on sow litter performance (means ± SD).

**Item**	**Control**	**Sildenafil**
No. of litters	42	51
Piglets born, no./litter	11.09 ± 2.28	11.94 ± 2.17^*^
Piglets born alive, no./litter	10.93 ± 2.05	11.82 ± 2.12^*^
Piglets weaned, no./litter	9.91 ± 1.56	10.89 ± 1.92^*^
Stillborn, no./litter	0.2	0.11
Mummies, no./litter	0.07	0.02
Litter birth weight, kg	16.93 ± 3.03	18.38 ± 3.59^*^
Piglet average birth weight, kg	1.49 ± 0.21	1.59 ± 0.29

* *p < 0.05*.

### Effect of Sildenafil Intervention From Gestation day 30 to 110 on the Litter Performance of Gilts

Next, we assessed the beneficial effect of dietary sildenafil supplementation during mid-to-late gestation is common in gilts. To this end, 19 gilts were given the basic diet supplemented with sildenafil (0.32 g/day) from GD 80 to GD 110. Gilts in the sildenafil-supplemented group had more piglets born alive than those in the control group. In addition, the litter birth weight and the average birth weight were higher in the sildenafil group than in the control group (Table 6). Thus, we concluded that dietary sildenafil supplementation is helpful in increasing the litter size of gilts.

**Table 6:** Effect of sildenafil intervention from day 30 to 110 of gestation on gilt litter performance (means ± SD).

**Item**	**Control**	**Sildenafil**
No. of litters	22	19
Piglets born, no./litter	10.72 ± 2.21	11.00 ± 1.41
Piglets born alive, no./litter	9.59 ± 2.68	11.00 ± 1.41^*^
Piglets weaned, no./litter	9.13 ± 1.26	10.00 ± 1.20
Stillborn, no./litter	0.45	0^*^
Mummies, no./litter	0.59	0
Litter birth weight, kg	16.12 ± 3.45	18.19 ± 2.16^*^
Piglet average birth weight, kg	1.51 ± 0.28	1.67 ± 0.18^*^
Litter weaning weight, kg	53.91 ± 7.84	57.24 ± 10.12
Piglet average weaning weight, kg	5.93 ± 0.60	6.86 ± 4.26

* * p < 0.05*.

## Discussion

The reproductive performance of sows and gilts and the growth performance of neonatal piglets are the critical determinants of pig farm management. Given that pigs are multiple ovulating species, uterine status is one of the most important limiting factors that determine litter size, having a vital impact on the growth performance of neonatal piglets. It has been reported that approximately 30–50% of released oocytes fail to survive (4, 5). The acquisition of sufficient nutrients and oxygen *via* placental blood flow is a prerequisite for embryo survival. Thus, stimulating uterine blood flow or improving vascular development or function is thought to be a promising strategy to enhance the reproductive performance of sows and gilts. Previous studies have reported that dietary supplementation with N-carbamylglutamate, which improves placental vascular function and promotes the nutrient supply to the fetus (20), during the entire gestation period, significantly improves the reproductive performance of gilts (21). Sildenafil citrate is a type 5-specific PDE inhibitor that prevents cGMP hydrolysis and potentiates the effect of NO on vascular smooth muscle. Therefore, sildenafil can effectively improve uterine arterial blood flow (13, 16), which in turn enhances fetal development or reproductive performance (16–18, 22, 23).

In our study, dietary supplementation with sildenafil from day 0 to 30 of gestation significantly improved the litter size of sows. A possible explanation is that sildenafil intervention during early gestation covers the critical period of embryo implantation. Based on results from previous studies, 20–45% of pig conceptuses would be lost after fertilization (23). Among these, 20–30% of pregnancy loss occurs during the peri-implantation period (day 12–30 of gestation) (5, 24, 25); while 10–15% of pregnancy loss occurs from mid-to-late gestation (GD 50–90) (26, 27). In addition, the period from mid- to late-gestation is also essential for fetal growth and is thus critical for the viability of the neonatal piglet. Therefore, dietary supplementation with sildenafil from GD 30 to 80 of gestation, as well as from GD 30 to 110, significantly improved the number of piglets born alive, probably because of the improved maternal-fetal nutritional transport from mid-to-late gestation. However, dietary supplementation with sildenafil specific to late gestation, i.e., from GD 80 to 110, did not improve the number of piglets born alive to gilts, implying that the number and viability of neonatal piglets were largely determined before the last month of gestation in gilts. Of course, it is likely that gilts receiving supplementation during the whole gestation period, i.e., from GD 0 to 110, would have more piglets born alive compared with gilts in the control group. Surprisingly, we found an interesting result that sows fed sildenafil supplementation from the mid-to-late gestation period experienced significantly improved litter performance, achieving levels comparable with those in the sows supplemented with sildenafil throughout the whole gestation period. This can be explained by the fact that early embryonic development is largely independent of maternal support, until the formation of a functional placenta. After placentation, dietary sildenafil supplementation might increase uterine blood flow and the transport capacity, thus improving maternal-fetal exchange. Therefore, sows supplemented with dietary sildenafil from mid-to-late gestation (GD 30–110) showed a significantly improved litter performance, to levels comparable with those in the sows treated throughout the whole gestation.

In addition to litter size, piglet birth weight is also an important index of the litter performance of sows and gilts. Previous studies have shown that an increase in litter size was associated with a reduction in piglet birth weights (22, 28). It has been estimated that the greatest proportion of mortality in commercial pig production occurs before weaning (29). Among the factors that cause preweaning mortality, lower piglet birth weight is one of the most important contributors (30). Piglets with low-birth weight have an increased risk of stillbirth (31, 32) and pre-weaning mortality (32–34). In our study, dietary supplementation with sildenafil from day 0 to 30, as well as from day 30 to 80 of gestation, did not affect piglet birth weight. By contrast, sildenafil supplementation from mid-to-late gestation, i.e., from day 30 to 110 of gestation significantly improved piglet birth weight, implying that uterine blood flow or vascular development/function during this period is critical for prenatal fetal growth. Feeding sildenafil throughout gestation also significantly increased the birth weight of the piglet.

It is worth mentioning that gilts showed a more notable increase in piglet birth weight compared with sows when sildenafil was supplemented from day 30 to 110 of gestation. The weight of the offspring from gilts is usually lower at birth (35, 36) and weaning (37, 38) than those from older parity gilts. The poorer growth performance of gilt offspring compared with sow offspring remains poorly understood. However, it is believed to be influenced by a number of factors, such as lower birth weight, poor colostrum and/or milk intake (39), and insufficient transfer of maternal immunoglobulins (40–42), suggesting the poorer uterine condition of gilts compared with that of sows. Dietary supplementation with sildenafil during gestation from day 30 to 110 might improve the uterine condition to achieve better litter performance comparable with that of sows. Moreover, the cost of sildenafil was $0.03 per pig per day. The low cost of sildenafil makes it a viable method for improving the reproductive performance of sows and gilts.

In conclusion, the results of the present study suggest that dietary supplementation with sildenafil during gestation has a notable beneficial effect on the reproductive performance of both sows and gilts, thus providing a promising and practical intervention strategy to improve reproductive management in pig farming.

## Data Availability Statement

The original contributions presented in the study are included in the article/supplementary material, further inquiries can be directed to the corresponding author.

## Ethics Statement

The animal study was reviewed and approved by Institutional Animal Care and Use Committee at the China Agricultural University (Beijing, China).

## Author Contributions

JT, LA, and YQ contributed to the study design. YW, FY, XH, WW, and LC carried out the experiments. WZ and YW contributed to the statistical analysis of the data. YW wrote the manuscript. All authors read and approved the final manuscript.

## Funding

This work was supported by grants from the National Key R&D Program (Grants 2017YFD0501901 and 2017YFD0501905) and the Beijing Innovation Consortium of Agriculture Research System.

## Conflict of Interest

The authors declare that the research was conducted in the absence of any commercial or financial relationships that could be construed as a potential conflict of interest.

## Publisher's Note

All claims expressed in this article are solely those of the authors and do not necessarily represent those of their affiliated organizations, or those of the publisher, the editors and the reviewers. Any product that may be evaluated in this article, or claim that may be made by its manufacturer, is not guaranteed or endorsed by the publisher.
